# Grammar acquisition in preschool children is related to white matter maturation of the dorsal language network

**DOI:** 10.1016/j.dcn.2026.101715

**Published:** 2026-03-22

**Authors:** Cheslie C. Klein, Philipp Berger, Charlotte Grosse Wiesmann, Angela D. Friederici

**Affiliations:** a Department of Neuropsychology, Max Planck Institute for Human Cognitive and Brain Sciences, Leipzig, Germany; b Research Group Milestones of Early Cognitive Development, Max Planck Institute for Human Cognitive and Brain Sciences, Leipzig, Germany; c Cognitive Neuroscience Lab, Department of Liberal Arts and Sciences, University of Technology Nuremberg, Germany

**Keywords:** Language development, Grammar acquisition, Morpho-syntax, White matter, Arcuate fascicle, Language network

## Abstract

In preschool years, children take important steps in grammar acquisition, which are essential to learning their native language. A central aspect is the acquisition of the morpho-syntactic rule system, which forms an intersection between words and sentences. In adults, rule-based linguistic processes are supported by the dorsal fiber pathway to BA44, the arcuate fascicle. This pathway matures late in development, raising the question of whether it already supports grammar processes in the early preschool years, or whether early grammar acquisition is supported by different, earlier-maturing fiber pathways. In a large sample of 3- to 5-year-old children (N = 120), we examined the association between maturation of the language network and children’s morpho-syntactic abilities. This revealed consistent differences between 3-year-olds and 4- to 5-year-olds. The 4- to 5-year-olds, but not 3-year-olds, showed a relation of morpho-syntax with both the dorsal pathway to BA6, supporting phonological processes, and the dorsal pathway to BA44, supporting syntactic processes in adults. Additionally, although less consistent, a relation with the ventral pathway, supporting semantic processes, was found in the 4- to 5-year-olds. Our results suggest that maturation of the language network enhances the acquisition of morpho-syntactic rules in the preschool period from 4 years of age.

## Highlights

• The dorsal language network supports grammar acquisition in the preschool years.
• The network includes the arcuate fascicle to BA44 and a second dorsal tract to BA6.
• These tracts support grammar in 4- to 5-year-old children but not in 3-year-olds.

## Graphical Abstract

**Figure:**
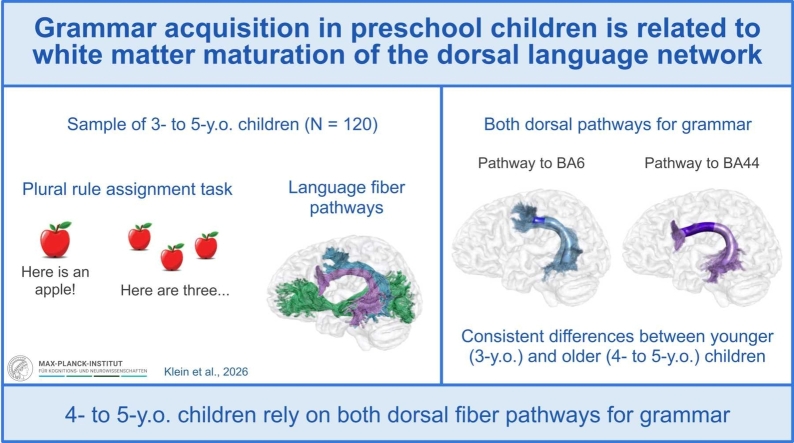

## 1 Introduction

Grammar is at the core of human language. When acquiring their native language, young children are faced with a difficult task. Not only do they need to learn a large number of new words from their input. They also need to extract rules governing the combination of words in a sentence as well as the combination of morphological elements of a word. It is an open question how the developing brain supports these processes during language acquisition.

While words contain the meaning, grammar rules set the relation between these words in a sentence, thereby allowing the formation of complex thoughts beyond the word-level. At the word-level grammatical rules guide the combinatorial processes of morphological elements in a word. This is especially evident in inflectional morphology, where the word stem and inflection are typically combined according to a set of rules. For example, the plural form of a noun takes on a more complex structure (e.g., dog-s) than the singular noun stem (e.g., dog) by combining it with a plural morpheme (e.g., -s). Morpho-syntax builds a bridge between the structure of the sentence and its words by modulating the grammatical features of a given word in relation to the sentence. Even though the combinatorial process is on the word-level, it enables the production and comprehension at the sentence-level (Harley, 2015).

During development, the ability to process rule-based regularities in auditory sequences is present in the first months of life, reflecting statistical learning of phonologically encoded regularities (Friederici et al., 2011, Marcus et al., 1999). Sentence-level syntactic rules are detected by the age of 2.5 years (Oberecker et al., 2005, Oberecker and Friederici, 2006). By 3 years of age, children can already produce and understand a variety of sentences (Kauschke, 2012, Schipke et al., 2012, Skeide et al., 2016, Strotseva-Feinschmidt et al., 2019), indicating that basic combinatorial processes seem to be in place by that age. However, milestones of complex grammar acquisition are only reached later, between the ages of 4–5 years and beyond (Crain and Thornton, 2012, Skeide and Friederici, 2016). Thus, the preschool years are considered as a take-off phase towards more complex grammar (Crain and Thornton, 2012, Fox and Grodzinsky, 1998, Guasti, 2002, Kauschke, 2012, Tomasello and Brooks, 1999).

At the neural level, the language network differs considerably between the developing and the adult brain. In adults, the neural language network consists of dedicated language regions in the inferior frontal cortex and the posterior temporal cortex known as Broca’s and Wernicke’s areas and the dorsally and ventrally located white matter fiber pathways interconnecting these regions (Friederici and Gierhan, 2013). In adults, grammatical processes involve BA44 in the posterior part of Broca’s area in the left inferior frontal gyrus in interaction with the posterior temporal lobe on the level of words (Bulut, 2022, Leminen et al., 2019, Meykadeh et al., 2023, Ullman, 2001) as well as sentences (Goucha and Friederici, 2015, Grodzinsky et al., 2021, Zaccarella et al., 2017). The neural correlates of inflectional morphology, which relies on rule-based processes, seem to be particularly related to activation in the left inferior frontal gyrus. In contrast, derivational morphology, which involves lexical meaning (e.g., happy vs. unhappy), seems to be more consistently processed in the temporal lobe (see for a review Leminen et al., 2019). The dorsal fiber pathway that connects these two brain regions, referred to as arcuate fascicle, has been suggested to support complex grammar in adults (Friederici and Gierhan, 2013, Griffiths et al., 2013, Wilson et al., 2011) including morpho-syntactic processes (Rolheiser et al., 2011). The ventral pathway, via the inferior fronto-occipital fascicle (IFOF), is proposed to be involved in semantic processes such as lexical access (Fridriksson et al., 2016, Friederici and Gierhan, 2013, Hickok and Poeppel, 2004, Rolheiser et al., 2011, Saur et al., 2008). The arcuate fascicle, dorsally connecting to BA44, can be distinguished from a second dorsal fiber bundle that connects the auditory cortex in the superior temporal lobe and BA6 in the premotor cortex. This latter pathway has been suggested to be involved in auditory-to-motor mapping as, for example, in speech repetition in adults (Fernández-Miranda et al., 2015, Friederici and Gierhan, 2013, Saur et al., 2008) and babbling in infants (Hickok and Poeppel, 2004).

Developmental brain studies have reported functional changes in the allocation of the language-related brain regions (Klein et al., 2023, Skeide et al., 2014) as well as maturational changes of the different fiber tracts (Skeide et al., 2016). A functional shift from involvement of the temporal cortex to the inferior frontal cortex has been observed between 3 and 4 years of age for sentence-level grammar processes (Klein et al., 2023). This shift towards more mature brain regions might not only underly the behavioral milestones in grammar between 3 and 4 years but might also point to alternative processing strategies for basic combinatorial processes before the age of 4 years. With respect to the fiber tracts, the dorsal pathway to BA6 and the IFOF are already detectable at birth, even though both fiber pathways still mature during childhood (Brauer et al., 2013, Perani et al., 2011). In contrast, the dorsal pathway to BA44 (i.e., the arcuate fascicle) could not be reconstructed in newborns when using a conservative fiber tracking method, suggesting a structurally less mature fiber pathway compared to the dorsal pathway to BA6 and the ventral fiber tract (Perani et al., 2011). Recent studies have successfully reconstructed the arcuate fascicle in newborns, however, without disentangling the two branches reaching into BA6 and BA44 (Grotheer et al., 2022, Grotheer et al., 2023). This has led to the hypothesis that early language development is predominantly supported by the dorsal pathway to BA6 and the IFOF, whereas the acquisition of complex sentence-level grammar from 4 years of age might require a more matured dorsal fiber connection to BA44 (Eichner et al., 2024, Skeide et al., 2016, Skeide and Friederici, 2016). This leaves open the question of how the maturation of the core language fiber pathways supports grammar acquisition on the word-level (i.e., morpho-syntax) in preschool-aged children.

The present study aims to clarify how the use and acquisition of morpho-syntactic rules relate to the maturation of core fiber connections of the language network in preschool years. We therefore investigated the association between developmental change in the dorsal and ventral fiber pathways of the language network and children’s grammar proficiency at the word-level during the critical preschool period of 3–5 years. For this, we analyzed preschooler’s morpho-syntactic performance in relation to structural properties of the two dorsal pathways targeting BA44 and BA6, and the ventral pathway (i.e., the IFOF), as well as a control tract, the corticospinal tract. This was investigated a large sample of 3- to 5-year-old children (N = 120), covering the ages where the shift in cortical brain regions and behavioral milestones in morpho-syntax have been observed. We hypothesized that children’s grammar abilities would be associated with indices of white matter maturation in the dorsal fiber pathway to BA44. In addition, we expected different patterns in this association between the 3-year-old versus the 4- to 5-year-old children, with younger children still relying on the well-matured dorsal pathway to BA6 or the IFOF.

## 2 Methods

The analysis pipeline and exclusion criteria were preregistered at https://aspredicted.org/FWL_62H.

### 2.1 Participants

For this study, MRI and behavioral data from 3- to 5-year-old monolingual German-speaking preschool children were analyzed (N = 120, mean age = 4.55, SD = 0.98, range = 3.07–6.16, 55 female; 3-y.o.: N = 47, mean age = 3.53, SD = 0.28, range = 3.07–3.99, 20 female; 4- to 5-y.o.: N = 73, mean age = 5.21, SD = 0.65, range = 4.01–6.16, 35 female). The sample was drawn from two previously collected samples (see Supplementary Material for further information on each sample). Children had no reported history of medical, neurological, or psychiatric disorder and no hearing or vision deficit.

The sample was a subsample from a larger behavioral data set of N = 281 children aged 3–5 years. The behavioral sample included children who provided data from a standardized test battery of general language development (SETK 3–5; *Sprachentwicklungstest für drei- bis fünfjährige Kinder: SETK3–5*; Grimm, 2001), independent of whether they also underwent MRI scans. Of these, N = 97 did not participate in or aborted the MRI. Further, children were excluded if they did not provide behavioral data of any relevant SETK 3–5 subtest (N = 1) or showed an indication for a speech development delay in the SETK 3–5 (T-value < 35; N = 10). Children were excluded from the MRI analyses if they received a later diagnosis of ADHD or developmental dyslexia (N = 16) or did not provide handedness data (N = 14). After MRI preprocessing, children were further excluded if they showed severe motion artifacts in the MRI scans (see *MRI preprocessing*; N = 16), or for which brain extraction, estimation of gradient direction or tractography failed (see *MRI preprocessing*; N = 8). For the standardization of the behavioral scores, children were further excluded from the initial behavioral sample if they did not provide data of all SETK 3–5 subtests (N = 18). This resulted in N = 252 children aged 3–5 years for standardization.

Parental consent was obtained for all children and the study was approved by the Ethics Committee at the Faculty of Medicine of the University of Leipzig (number of approval; Sample 1: 320–11–26092011, Sample 2: 090/12-ff). Data of both samples have previously been analyzed separately with regard to other research questions (Sample 1: Eichner et al., 2024; Kuhl et al., 2020, Kuhl et al., 2021; Liebig et al., 2020; Qi et al., 2021; Sample 2: Berger et al., 2021; Grosse Wiesmann et al., 2020; Grosse Wiesmann, Friederici, et al., 2017; Grosse Wiesmann, Schreiber, et al., 2017; Klein et al., 2023).

### 2.2 Behavioral data

#### 2.2.1 Morpho-syntactic ability

The SETK 3–5 is a comprehensive diagnostic tool designed to test 3- to 5-year-old children’s general language development in the domains of language comprehension, production, and memory (Grimm, 2001). To test children’s grammar abilities on the word-level, we selected and preregistered the morpho-syntactic word production task (SETK 3–5 subtest ‘Morphologische Regelbildung’; Grimm, 2001), which determines the acquisition level of the morpho-syntactic rule system for German plural formation.

In this task children are asked to name the plural of given nouns. For this, children are first shown a picture of an object or animal that is described using the singular form of a noun (e.g., “Schau mal, hier ist ein Auto.” [engl. “Look, here is a car.”]; Grimm, 2001). Then, a picture with three of the same objects or animals is shown and the child is asked to produce the plural form of the given word (e.g., “Hier kommen noch mehr dazu. Hier sind drei…?” [engl. “Here are some more. Here are three…?”]; Grimm, 2001). Children are familiarized with the task with one practice item and then tested on ten common real nouns, which follow the various dominant plural rules of the German noun system (for further description of the German plural system see Supplementary Methods). The 4- to 5-year-old children were presented, in addition, with eight pseudo words similar to the Wug Test from Berko (1958) (for a list of the items see Supplementary Table 1; Grimm, 2001). The age groups were determined based on which version of the SETK 3–5 was performed as the version for the 3-year-old children slightly differed (i.e., real nouns) from the version for the 4- to 5-year-old children (i.e., real and pseudo nouns).

Children’s responses for each item were rated depending on whether a plural rule was used, and which one. A score of 2 was given for the correct plural form, of 1 for the use of a different but incorrect plural rule (e.g., “Fisch-en” instead of “Fisch-e”; Grimm, 2001). A score of zero was given for an incorrect response, for example, repetition of the singular form or a plural noun with significant changes of the word stem (e.g., “Gratel-en” instead of “Gabel-n”; Grimm, 2001), or no response at all. In the present study, we used raw values of the morpho-syntactic word production task rather than normalized scores provided by the SETK 3–5, as we were interested in relating individual developmental differences to brain structure. To account for the differences in item structure among 3- and 4- to 5-year-olds, we z-transformed these raw scores within each age group. The standardization was carried out within the full behavioral datasets of each sample (N = 252). The resulting morpho-syntax score was used for the brain-behavior analyses.

In addition, we preregistered a syntactic comprehension and production score from two further subtests of the SETK 3–5 to test for children’s grammar ability on the sentence-level, which we report in the Supplementary Material. These additional grammar scores were added to investigate the relation between children’s grammar ability at the sentence-level (i.e., syntax) as opposed to the word-level (i.e., morphology), which was tested by the morpho-syntax scores. Exploratory analyses revealed that syntactic comprehension scores did significantly correlate with children’s morpho-syntax scores across both age groups (*r*(117) = 0.27, *p* = 0.003). Syntactic productions scores were also significantly correlated to children’s morpho-syntax scores (*r*(113) = 0.37, *p* < 0.001). Further, syntactic comprehension and syntactic production scores were significantly related (*r*(114) = 0.40, *p* < 0.001).

#### 2.2.2 Psychometrics

Children’s non-verbal IQ was assessed using the Wechsler Preschool and Primary Scale of Intelligence (WPPSI-III; Wechsler, 2009) in Sample 1, and the Kaufman Assessment Battery for Children (Kaufman ABC; Melchers and Preuß, 2003) in Sample 2. Children’s non-verbal IQ scores were within the typical range for the respective age: For Sample 1, the standard scores of the non-verbal scale were used (mean = 98.33, SD = 13.30), and for Sample 2, the mean scale scores were used (mean = 10.26, SD = 1.34). To ensure comparison across samples, both scores were standardized within each sample. Children’s degree of handedness was evaluated with the German version of the Edinburgh Handedness Inventory (Oldfield, 1971).

### 2.3 MRI data acquisition

MRI data were obtained on a 3 T Siemens scanner (Siemens MRT Trio series) equipped with a 12-channel head coil for Sample 1 and a 32-channel head coil for Sample 2.

In Sample 1, diffusion-weighted MRI (dMRI) data were acquired using the optimized monopolar Stejskal-Tanner EPI sequence (Morelli et al., 2010) along the anterior-to-posterior phase encoding (PE) direction (voxel size = 1.86*×*1.86*×*1.9 mm, TR = 8000 ms, TE = 83.0 ms, b-value = 1000 s/mm^2^, 60 directions, GRAPPA 2, acquisition time = 9:20 min). A field map was acquired after completion of each 10 images. In addition, one diffusion-weighted image and a field map with b = 0 s/mm^2^ was acquired along the posterior-to-anterior PE direction. In Sample 2, dMRI data were acquired using the multiplexed echo planar imaging (EPI) sequence (Feinberg et al., 2010) with a spatial resolution of 1.9 mm isotropic (TR = 4000 ms, TE = 75.4 ms, b-value = 1000 s/mm^2^, 60 directions, GRAPPA 2, acquisition time = 5:32 min). After the dMRI scan, a field map with b = 0 s/mm^2^ was acquired as an anatomical reference.

High-resolution 3D T1-weighted MRI data were further acquired in both samples using the MP2RAGE sequence (Marques et al., 2010) (Sample 1: voxel size = 1.3 mm isotropic, TR = 5,000 ms, TE = 2.82 ms, TI_1_ = 700 ms, α_1_ = 4°, TI_2_ = 2,500 ms, α_2_ = 5°, acquisition time = 6:22 min; Sample 2: voxel size = 1*.*2*×*1.0*×*1.0 mm, TR = 5000 ms, TE = 3.24 ms, TI_1_ = 700 ms, α_1_ = 4°, TI_2_ = 2500 ms, α_2_ = 5°, acquisition time = 5:22 min).

A few days before the actual MRI scan, children were familiarized with the environment and the procedure by performing a mock scan in a playful setting. During the actual MRI scan, children watched a movie of their choice on MRI-compatible goggles and headphones to ensure children’s comfort and achieve better data quality.

A summary table of the MRI parameters applied in the two samples can be found in the Supplementary Material (see Supplementary Table 2).

### 2.4 MRI data analysis

#### 2.4.1 MRI preprocessing

MRI preprocessing of the dMRI data included the following steps (for Sample 1 see Eichner et al., 2024; Fan et al., 2022; for Sample 2 see Grosse Wiesmann et al., 2017). In Sample 1, the underlying noise distribution was used to correct for non-Gaussian noise biases in the data (St-Jean et al., 2020). Further, data was denoised and Gibbs ringing artefacts were removed using MRtrix3’s *dwidenoise* and *mrdegibbs* (Kellner et al., 2016, Tournier et al., 2019, Veraart et al., 2016). Estimation of the susceptibility-induced off-resonance field was done using FSL’s *topup* tool to correct for distortions (Andersson et al., 2003, Smith et al., 2004). Motion and eddy current correction was applied with FSL’s *eddy* (Andersson and Sotiropoulos, 2016). We additionally performed signal drift correction using a second-order polynomial fit to the data without diffusion-weighting (Vos et al., 2017). In Sample 2, volumes corrupted by motion were automatically removed before preprocessing (see for details Schreiber et al., 2014). Motion and eddy currents were corrected by aligning the diffusion-weighted images to the last image without diffusion-weighting using FSL’s *flirt*. The field map was used to estimate distortions with FSL’s *prelude* and *fugue*. Diffusion-weighted data was further aligned to the preprocessed T1-weighted data (see below for details on the preprocessing). All transformations were applied in a single interpolation step to the data using FSL’s *applywarp*.

T1-weighted MRI data were preprocessed with the following steps. Background noise was removed by combining the two inversion time images (O’Brien et al., 2014). In Sample 1, the fsl_anat pipeline was then used to reorient and register the data to the MNI space, perform brain extraction, and segment the brain tissues (http://fsl.fmrib.ox.ac.uk/fsl/fslwiki/fsl_anat). Then, the data was aligned to ACPC using the Automatic Registration Toolbox (https://www.nitrc.org/projects/art). Finally, diffusion data were aligned to the processed T1-weighted images following the *dtiInit* pipeline from the VISTASOFT package (https://github.com/vistalab/vistasoft). In Sample 2, the same preprocessing steps were performed using SPM8 (https://www.fil.ion.ucl.ac.uk/spm/software/spm8/) and FSL’s *flirt* and *aff2rigid*.

After preprocessing of the diffusion- and T1-weighted MRI data, we performed several quality checks on the processed data. First, we used FSL’s *eddy QC* tool to perform quality assessment of diffusion data on the single-subject level (Bastiani et al., 2019). Based on the output of this tool, we excluded children with large head movement values, more than 2 SDs from each sample’s mean movement (N = 16). The absolute motion ranged between 0.23 and 2.04 mm (3-y.o.: mean = 0.83 mm, SD = 0.45 mm, median = 0.67, range = 0.26–2.04 mm; 4- to 5-y.o.: mean = 0.84, SD = 0.47, median = 0.69, range = 0.23–2.01; see Supplementary Figure 4 for the distribution of absolute motion within each age group). Further, a diffusion tensor model was fitted at each voxel using FSL’s *dtifit* and the eigenvectors of prominent tracts as the superior longitudinal fascicle, corpus callosum, and corticospinal tract were visually checked on the FA images. Note that we only used the fitted tensor models for visual inspection, not for later fiber tracking, which was based on the Constrained Spherical Deconvolution model (CSD; see below for details). Third, successful brain extraction was visually checked, both for the diffusion- and T1-weighted MRI data (N = 4 exclusions).

#### 2.4.2 Automated tractography pipeline

To reconstruct the left dorsal and ventral language-related fiber pathways and the corticospinal tract as a control from the preprocessed dMRI data, we used the python-based version of the Automated Fiber Quantification (pyAFQ 0.12) software (Kruper et al., 2021, Yeatman et al., 2012). This pipeline utilizes several steps to generate well-defined and anatomically plausible fiber pathways. Further, it results in global measures such as the streamline count of each pathway, as well as so-called tract profiles of local assessments of microstructural diffusion properties along each pathway (Kruper et al., 2021, Yeatman et al., 2012).

We performed the following steps as provided by the pyAFQ pipeline. The distribution of fiber orientation at each voxel was estimated using the CSD model (Tournier et al., 2004) implemented as the default in pyAFQ. A whole-brain probabilistic tractography was then performed within the default pyAFQ pipeline which relies on DIPY (Garyfallidis et al., 2014) with maximum turning angle = 30°, minimum streamline length = 10 mm, maximum length = 1000 mm, and step-size = 0.5 mm.

The resulting tractograms were then segmented into the three language-related fiber pathways of interest and the additional control tract by using predefined endpoint and waypoint ROIs, based on prior anatomical knowledge. For the endpoint ROIs, regions from the AAL atlas provided for use within the pyAFQ pipeline (Rokem, 2021, Tzourio-Mazoyer et al., 2002) were selected to define the cortical termination points of each fiber pathway. Waypoint ROIs were defined in MNI space and then registered to children’s native space using a non-linear transformation to further guide the segmentation to result in anatomically plausible pathways (Wakana et al., 2007). Within the pyAFQ pipeline, endpoint and waypoint ROIs for over 20 major fiber pathways are provided by default, including the arcuate fascicle, IFOF, and corticospinal tract. We relied on the default segmentation protocol of pyAFQ when possible. To segment the ventral fiber pathway, corresponding to the IFOF and the corticospinal tract as control tract, we relied on the default endpoint and waypoint ROIs provided by pyAFQ (see Supplementary Table 5 and for details Kruper et al., 2021; Yeatman et al., 2012). To disentangle the dorsal pathway to BA44 from the one to BA6, we modified the default segmentation pipeline for the arcuate fascicle as the default arcuate fascicle protocol does not distinguish its sub-segments (see Supplementary Figure 5 *and* Supplementary Table 5). We used the default endpoint ROI in the temporal lobe and the default waypoint ROIs of the arcuate fascicle, which were provided by the pyAFQ pipeline, for both dorsal fiber pathways. To identify the termination points of each dorsal pathway in BA44 and BA6, respectively (Frey et al., 2008, Glasser and Rilling, 2008), we modified the endpoint ROIs in the frontal cortex for each dorsal pathway. For the dorsal pathway to BA44, we used the AAL atlas label for BA44, and for the dorsal pathway to BA6, we used the BA6 label (see Supplementary Table 5 for label indices). To further ensure an accurate segmentation of the dorsal pathways to BA44 and BA6, we used additional waypoint ROIs from the Harvard-Oxford atlas (see Supplementary Table 5 for label indices; Makris et al., 2006). For the dorsal pathway to BA6, we further defined an exclusion ROI in BA44 to disentangle streamlines of both dorsal fiber pathways. To prevent fibers of the dorsal pathway to BA6 from branching into the corticospinal tract, we used the default endpoint mask of the corticospinal tract, provided by the pyAFQ pipeline, as a second exclusion ROI (see Supplementary Figure 5*)*.

The segmented fiber pathways were then further refined by comparing each candidate fiber to a probability map (Hua et al., 2008) and discarding fibers passing low probability regions. By default, pyAFQ provides probability maps for the arcuate fascicle, which was used for both dorsal fiber pathways, for the IFOF, and corticospinal tract. To ensure a compact definition of each fiber pathway, we used a more conservative probability threshold of 3 (compared to the pyAFQ default of 0). Outlier fibers that deviated substantially from the core of the respective fiber pathway were additionally removed to result in a compact fiber bundle. To identify the fiber core, each pathway was clipped at the endpoint ROIs, resampled into 100 equidistant nodes along the pathway and the mean location of each node was calculated. The resulting bundles of each child were visually inspected by the first author while being blind to the age of each child to ensure biologically plausible identification of each fiber pathway. Fiber bundles of the two dorsal fiber pathways to BA44 and BA6 were excluded if streamlines followed anatomically implausible turns, such as ventrally forming a loop, if the reconstructed streamlines were visually very sparse and therefore likely spurious, or if the frontal endpoints of the bundles did not terminate in the defined areas of BA44 or BA6, but instead intertwined (for examples see Supplementary Figure 6). The visual inspection led to the exclusion of N = 4 of the dorsal pathway to BA44 (3-y.o.: N = 1; 4- to 5-y.o.: N = 3), N = 2 of the dorsal pathway to BA6 (3- y.o.: N = 1; 4- to 5- y.o.: N = 1), and N = 1 of the corticospinal tract of a 3-year-old child, and N = 1 of the IFOF of a 4- to 5-year-old child.

Diffusion properties (i.e., fractional anisotropy [FA], mean diffusivity [MD], and radial diffusivity [RD]) were quantified at each node along each fiber pathway using a weighted approach as described by Yeatman et al. (2012). This resulted in a so-called tract profile that was used for further analysis. In addition, total streamline count of each fiber pathway was calculated. As this measure is influenced by the curvature, length, and branching of the underlying fiber pathways, which makes its biological interpretation challenging (Jones et al., 2013), we report these preregistered analyses in the Supplementary Material.

We additionally reconstructed the homologous dorsal and ventral language pathways and the corticospinal tract as a control in children’s right hemisphere. These exploratory analyses are reported in the Supplementary Material.

#### 2.4.3 Statistical analysis

To estimate the relation between children’s morpho-syntactic ability and structural properties of the language-related fiber pathways and the control tract, we fit multiple linear regressions using the lm() function, which performs ordinary least squares regression, from the stats package included in R (R Core Team, 2023) using RStudio (Posit team. 2024). We used the gvlma package to check if the assumptions of each linear regression were met (Peña and Slate, 2006). As preregistered, we used the morpho-syntax scores as the predictor and the brain structural measures (i.e., FA, MD, and RD) as dependent variables in each model. We included both age groups in the analyses, with age group as factor and tested for a main effect and an interaction between age group and children’s morpho-syntax scores. Significant interactions with age group were followed up in each age group separately. In addition, for the purpose of comparison, we also explored language scores with a nonsignificant age interaction in the separate age groups. In all models, we controlled for sex, non-verbal IQ, handedness, and estimated intracranial volume (eTIV) to examine the specificity of the effects. As Sample 1 consisted of children with and without a family history of dyslexia, we included family history as an additional covariate in the analyses. Further, as the MRI acquisition parameter differed between the two samples, ‘sample’ was included as a covariate of no interest. In all models testing for a main effect, we performed one-sided tests as a strong body of evidence indicate that FA increases with maturation and higher cognitive function, while MD and RD decrease (Lebel et al., 2019, Lebel and Deoni, 2018, Schmithorst and Yuan, 2010, Yoshida et al., 2013). Multiple linear regressions were performed pointwise along 100 segments (i.e., nodes) of each fiber pathway. We therefore applied cluster-wise correction for multiple comparisons at *p* = 0.05 using non-parametric permutation-based correction (Nichols and Holmes, 2002) with a further Bonferroni correction for the N = 3 microstructural indices (i.e., for FA, MD, and RD). Statistics for the cluster-wide relationship between these indices and morpho-syntax scores were calculated on the mean FA, MD, or RD of the respective significant cluster. We winsorized all language data and brain maturational indices limiting extreme values to the 95th percentile prior to conducting analyses.

To relate brain maturational indices of the language-related fiber pathways to preschooler’s grammar performance on the sentence-level, we performed similar analyses on children’s syntactic comprehension and production scores as reported in the Supplementary Material.

## 3 Results

**Fig. 1:**
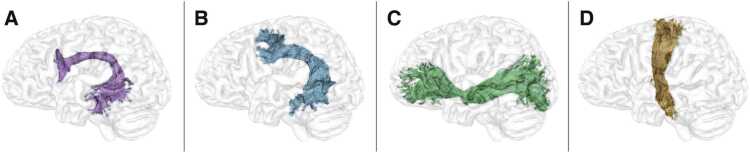
Example of the segmented and refined language fiber pathways – A) the dorsal pathway to BA44 (purple), B) the dorsal pathway to BA6 (blue), C) the ventral pathway (green) – and D) the corticospinal tract as control tract (orange).

To investigate the relation between 3- to 5-year-old preschooler’s abilities in morpho-syntactic processing and brain structural maturation of language-related fiber pathways, we reconstructed two dorsal fiber pathways, one targeting BA44 and one targeting BA6, as well as the ventral pathway (i.e., IFOF), and the corticospinal tract as a control tract (see Fig. 1). In a preregistered procedure, we then related their brain maturational indices (i.e., local measures of FA, MD, and RD along 100 nodes of each pathway) to children’s morpho-syntactic abilities, which were assessed using a noun plural assignment task. All effects were independent of sex, non-verbal IQ, handedness, eTIV, and, additionally independent of family history of dyslexia and sample. The assumptions of the linear regression were met for each node of all reported clusters. Multiple comparison correction was applied at *p* = 0.05 with a further Bonferroni-correction to correct for N = 3 brain maturational indices. Reported statistics for the cluster-wide relationship between brain maturational indices and morpho-syntax scores were calculated on the mean FA, MD, or RD of the respective significant cluster after applying multiple comparison correction.

**Fig. 2:**
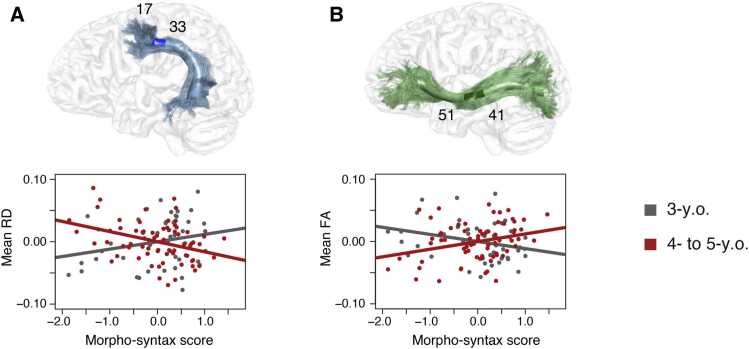
Linear partial correlation of preschooler’s morpho-syntactic abilities and brain structural measures within the significant clusters of the age interactions after controlling for sex, non-verbal IQ, handedness, eTIV, and additionally for family history of dyslexia and sample. Cluster-wise correction was applied at *p* = 0.05 with a further Bonferroni-correction of N = 3 to identify significant interactions. Residuals of the partial correlation of the morpho-syntax score and the mean values of the brain maturational measures within the significant clusters are plotted. A) Significant age interaction with radial diffusivity (RD) and morpho-syntax scores in the dorsal pathway to BA6 (blue; 3-y.o.: *r*_*p*_ = 0.24, gray; 4- to 5-y.o.: *r*_*p*_ = −0.36, red). B) Significant age interaction with fractional anisotropy (FA) and morpho-syntax scores in the ventral pathway (green; 3-y.o.: *r*_*p*_ = −0.29, gray; 4- to 5-y.o.: *r*_*p*_ = 0.28, red).

In these analyses, younger children at the age of 3 years differed from older children aged 4–5 years. There was no significant main effect of children’s morpho-syntax scores on their brain maturational measures in the reconstructed pathways, but a significant interaction with age group. This age interaction was found in the dorsal pathway to BA6 (anterior part: RD, node range = 17–33, cluster size = 17, cluster threshold = 13, β = −0.027, SE = 0.008, F^2^ = 0.10, df = 107, *p* < 0.017 FWE-corrected; see Fig. 2a) and in the ventral pathway (central part: FA, node range = 41–51, cluster size = 11, cluster threshold = 11, β = 0.025, SE = 0.007, F^2^ = 0.11, df = 108, *p* < 0.017 FWE-corrected; see Fig. 2b), but not the dorsal pathway to BA44. No main effect or interaction with age was found in the corticospinal tract serving as control tract. To follow-up on these interactions, we analyzed the two age groups, i.e. the 3- and 4- to 5-year-olds, separately. This revealed that the interaction in the dorsal pathway to BA6 was driven by the older children who showed a significant relation between their morpho-syntax scores and brain maturational measures in the same portion of the tract (anterior part: RD, node range = 17–33, cluster size = 17, cluster threshold = 14, β = −0.016, SE = 0.005, F^2^ = 0.11, df = 63, *p* < 0.017 FWE-corrected). For the ventral pathway, only a relation between total streamline count and morpho-syntax scores was observed in the 4- to 5-year-old children (as reported in the *Supplementary Results*). No effect was found in the dorsal pathway to BA44 or the control tract in the 4- to 5-year-olds. The 3-year-olds did not show any significant relation in either of the three language-related fiber pathways or the control tract.

In order to test whether these correlations between behavior and white matter fiber tracts were specific for the left hemisphere, we also analyzed the corresponding white matter fiber tracts in the right hemisphere. For the right-hemispheric homologous fiber pathways, we found no main effect, but a significant age interaction, which was driven by the 4- to 5-year-olds in the right ventral pathway, for children’s morpho-syntax scores. No main effect was found for the right dorsal pathway to BA44 or the right dorsal pathway to BA6 in the older age group, but a significant interaction with age group (see Supplementary Results for more details).

**Fig. 3:**
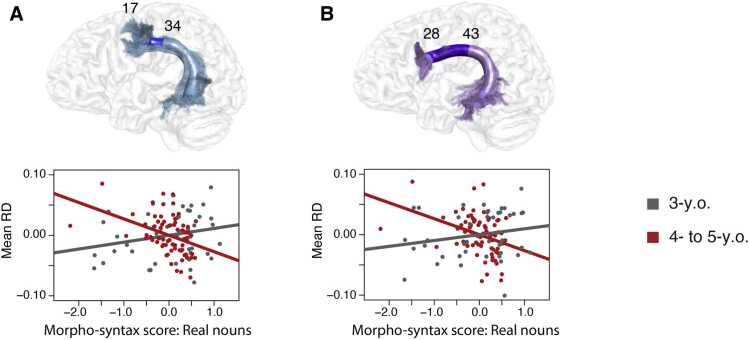
Linear partial correlation of preschooler’s morpho-syntactic abilities tested with real nouns and brain structural measures within the significant clusters of the age interactions after controlling for sex, non-verbal IQ, handedness, eTIV, and additionally for family history of dyslexia and sample. Cluster-wise correction was applied at *p* = 0.05 with a further Bonferroni-correction of N = 3 to identify significant interactions. Residuals of the partial correlation of the morpho-syntax score tested with real nouns and the mean values of the brain maturational measures within the significant clusters are plotted. A) Significant age interaction with radial diffusivity (RD) and morpho-syntax scores tested with real nouns in the dorsal pathway to BA6 (blue; 3-y.o.: *r*_*p*_ = 0.23, gray; 4- to 5-y.o.: *r*_*p*_ = −0.40, red). B) Significant age interaction with radial diffusivity (RD) and morpho-syntax scores tested with real nouns in the dorsal pathway to BA44 (purple; 3-y.o.: *r*_*p*_ = 0.19, gray; 4- to 5-y.o.: *r*_*p*_ = −0.36, red).

Further, to test whether the interaction with age found in our analyses could be due to the fact that the noun plural assignment task in 3-year-olds only contained real nouns, while in 4- to 5-year-olds additionally contained pseudo nouns, an analysis only including the real nouns was conducted. This was done as the generation of plural forms of real nouns and pseudo nouns might be based on different strategies since the plural forms of pseudo nouns cannot be retrieved from the mental lexicon but requires rule application. Thus, in an exploratory analysis, we divided the items presented to the 4- to 5-year-olds into real and pseudo nouns. The summed score of real noun items were standardized across both age groups. We then tested for a relation between children’s real noun morpho-syntax scores and maturational indices in the language pathways and the control tract. For children’s real noun morpho-syntax scores, as before, no significant main effect but significant interactions with age group were found. These interactions were found in the dorsal pathway to BA6 (anterior part: MD, node range = 7–32, cluster size = 26, cluster threshold = 20, β = −0.026, SE = 0.008, F^2^ = 0.11, df = 107, *p* < 0.017 FWE-corrected; RD, node range =17–34, cluster size = 18, cluster threshold = 13, β = −0.037, SE = 0.011, F^2^ = 0.12, df = 107, *p* < 0.017 FWE-corrected; see Fig. 3a), and in the dorsal pathway to BA44 (anterior to central part: RD, node range =28–43, cluster size = 16, cluster threshold = 12, β = −0.037, SE = 0.011, F^2^ = 0.10, df = 105, *p* < 0.017 FWE-corrected; see Fig. 3b). As before, follow-up analyses revealed that the present interactions were driven by the older age group. The 4- to 5-year-olds, but not the 3-year-olds, showed an effect in the dorsal pathway to BA6 (anterior part: MD, node range = 5–35, cluster size = 31, cluster threshold = 22, β = −0.022, SE = 0.006, F^2^ = 0.21, df = 63, *p* < 0.017 FWE-corrected; RD, node range = 17–34, cluster size = 18, cluster threshold = 14, β = −0.027, SE = 0.008, F^2^ = 0.22, df = 63, *p* < 0.017 FWE-corrected) and in the dorsal pathway to BA44 (anterior to central part: MD, node range = 15–45, cluster size = 31, cluster threshold = 23, β = −0.019, SE = 0.006, F^2^ = 0.18, df = 61, *p* < 0.017 FWE-corrected; RD, node range = 27–44, cluster size = 18, cluster threshold = 13, β = −0.026, SE = 0.008, F^2^ = 0.19, df = 61, *p* < 0.017 FWE-corrected). No effect was found for the ventral pathway. Analyses for the pseudo noun plurals can be found in the Supplementary Material.

These results indicate that the age difference between the 3-year-olds and the older children found for the entire set of nouns was not driven by different subsets of the experimental material. To further investigate if the observed differences between the 3-year-olds and the older age group (i.e., the 4- to 5-year-olds) were systematically influenced by absolute head motion during scanning, we conducted additional analyses. For this, we tested for the age interactions with the real noun morpho-syntax score while controlling for motion. These additional analyses revealed that the interactions remained significant, indicating they were not driven by motion (see Supplementary Results). Further, no significant difference in absolute motion was found between the 3-year-old children and the older age group as tested via a Wilcoxon rank sum test (W = 1721, *p* = 0.98). Additionally, we performed sensitivity analyses to investigate whether the smaller sample size of the 3-year-old children (N = 47 vs. N = 73 for 4- to 5-y.o.) was too small to detect an effect. These analyses revealed that the sample size of the 3-year-olds was likely large enough to detect effects similar to those observed in the older children (see Supplementary Results for details).

## 4 Discussion

The present paper investigated the relation between grammar acquisition at the morpho-syntactic level and brain maturation at preschool age. Children between 3 and 5 years of age are known to undergo major behavioral improvements in their morpho-syntactic performance (Kauschke, 2012). However, the dorsal fiber pathway to BA44 supporting grammar in adults matures relatively late during development (Brauer et al., 2013, Skeide and Friederici, 2016). This raises the question of whether this fiber pathway already supports morpho-syntax acquisition in the early preschool years, or whether earlier maturing brain structures take on this role. We investigated this question in 3- to 5-year-old children. Our analysis showed consistent differences in the association between the maturation of language fiber pathways and children’s morpho-syntactic abilities between the 3-year-olds and the older children. The 4- to 5-year-old children showed a relation of their morpho-syntax scores with the anterior part of the dorsal pathway to BA6. In an exploratory analysis, in which we analyzed this relation in real nouns only, we further found a relation of the dorsal pathway to BA6 and in addition the dorsal pathway to BA44 with morpho-syntax abilities in the 4- to 5-year-olds, but not in the 3-year-olds. These results reveal a clear difference of the involvement of the dorsal language pathways in the older compared to the younger children.

In the adult brain, the dorsal pathway to BA6 has been found to support auditory-to-motor mapping and phonological processes, such as in speech repetition (Saur et al., 2008). In the present study, we observed an association between 4- to 5-year-old children’s morpho-syntactic abilities and the maturation of this pathway. The plural rule generation task, which was assessed in the present production task, involved processing a given noun, applying the correct plural rule, and articulating the plural form. Based on its function in previous adult studies, the dorsal pathway to BA6 may support these processes, and thus a more mature connection between the auditory and premotor cortices may advance children’s task performance. Importantly, the involvement of the dorsal pathway to BA6 was consistent in the older age groups and independent of whether the presented nouns were real words or included pseudo nouns. This suggests that this fiber pathway may be generally involved in the different aspects of the plural noun assignment task, such as the processing of the given nouns, articulating the plural, and retrieving plural rules. In addition, we found a relation between children’s real noun morpho-syntax scores and the dorsal pathway to BA44. In the mature brain, this pathway supports rule-based language processes, as found both in adult and patient studies (Friederici and Gierhan, 2013, Griffiths et al., 2013, Wilson et al., 2011). In children, the maturation of the dorsal pathway to BA44 is related to sentence processing abilities from 4 years of age (Skeide et al., 2016). Preschool children evidently build up a rule system during the acquisition of plural nouns (Kauschke, 2012). This is apparent in the overgeneralization errors children produced that mainly follow frequent plural rules (see Supplementary Figure 2; Kauschke et al., 2011; Laaha, 2011; Szagun, 2001). In line with this interpretation, not only plural assignment to real nouns, but also to pseudo nouns, was associated with the dorsal pathway to BA44 in 4-year-olds in Sample 2. Thus, maturation of the dorsal pathway to BA44 might not only support the acquisition of syntactic processes at the sentence-level (Skeide et al., 2016) but also of rule-based processes on the word-level.

Further, we found an association in the ventral pathway – although only with streamline count – with 4- to 5-year-old’s morpho-syntactic ability for the combined score including real and pseudo nouns. This was driven by the pseudo words as we found a relation with 4- to 5-year-olds’ pseudo noun scores only, but not for the score with the real nouns. The ventral fiber pathway mainly supports semantic processes such as lexical retrieval (Fridriksson et al., 2016, Friederici and Gierhan, 2013, Hickok and Poeppel, 2004, Saur et al., 2008) and was proposed as associative system (Goucha et al., 2017). Studies in patients and adults suggest that the lexical features of a word are retrieved prior to morpho-syntactic assignment, which is reflected in an association with the ventral pathway (Rolheiser et al., 2011, Sahin et al., 2009, Yablonski et al., 2019, Yablonski et al., 2021). In the morpho-syntactic rule generation task, children might have performed better in the generation of the pseudo noun plurals by forming an association between the pseudo noun and a phonologically related existing noun retrieved from the lexicon to assign the plural (e.g., the pseudo noun ‘Ropf’ rhymes with the German noun ‘Topf’ [engl. ‘pot’]).

While the association with the ventral pathway was also observed in the right hemisphere, 4- to 5-year-old children’s morpho-syntactic ability related to the dorsal pathways only in the left hemisphere (as reported in the Supplementary Material). These findings are consistent with the left-lateralization of the dorsal language pathways and that children’s sentence comprehension performance increases with left-lateralization of the dorsal pathway to BA44 (Eichner et al., 2024, Lebel and Beaulieu, 2009). Further, in the present study, we found an association between grammar ability at the word-level (i.e., inflectional morphology) and language fiber pathways, but not with grammar at the sentence-level (i.e., syntax). In particular the dorsal pathway to BA44 still matures during middle childhood (Brauer et al., 2013) and has been shown to correlate with children’s ability to comprehend syntactically complex sentences throughout childhood (Skeide et al., 2016). Thus, we hypothesize that the association between grammar ability at the sentence-level and the structural maturation of the dorsal pathway to BA44 may stabilize after the preschool period.

Moreover, we consistently found differences in the association between morpho-syntax and white matter between 3-year-old compared to 4- to 5-year-old children. Analyses of each sample separately revealed that these differences already occurred between 3 and 4 years of age (see Supplementary Results). This mirrors findings in cortical brain structure, where different local brain regions supported syntax in 3- compared to 4-year-olds (Klein et al., 2023). These qualitative changes in brain structures – both in grey and white matter – might support the behavioral differences in grammar acquisition that have been found after 4 years of age, such as the acquisition of subordinate clauses and passive constructions (Crain and Thornton, 2012, Fox and Grodzinsky, 1998, Guasti, 2002, Kauschke, 2012, Tomasello and Brooks, 1999).

To summarize, we found a consistent association between 4- to 5-year-old children’s abilities to apply morpho-syntactic rules and the maturation of the dorsal language network, which was not found for the 3-year-old children. In adults, the dorsal connection between BA44 and the temporal cortex is crucial for rule-based processes, such as morpho-syntax (Meykadeh et al., 2023, Rolheiser et al., 2011), while the dorsal pathway to BA6 supports phonological processes as auditory-to-motor mapping (Friederici and Gierhan, 2013, Saur et al., 2008). Maturation of these brain structures might also support the acquisition of rule-based processes at the word-level in the preschool years.

## 5 Limitations

In the present study, we consistently found differences between the 3- year-old and the 4- to 5-year-old children. However, the 3-year-olds did not show any relation when tested individually. This highlights the need for further studies to clarify which brain structures are involved in grammar before the age of 4, which will be a significant challenge for future research given the difficulty of acquiring MRI data in young preschool children under 4 years of age. Further, here we investigated grammar ability at the word-level, i.e. morpho-syntax. An exciting avenue for future research is the question of which age the association between grammar ability on the sentence-level and brain structural maturation of the dorsal pathway to BA44 arises, given the ongoing maturation of this pathway even during middle childhood (Brauer et al., 2013, Skeide et al., 2016). Further, we tested children’s morpho-syntactic abilities with nouns. Given the different trajectories of nouns and verbs in language acquisition and the findings of dissociative neural correlates in adults for nouns versus verbs (Vigliocco et al., 2011, Waxman et al., 2013), future research should investigate whether the present findings also hold for the morpho-syntactic inflection of verbs. Moreover, we focused on the core language network including the two dorsal tracts targeting BA44 and BA6, and the ventral tract (Friederici and Gierhan, 2013, Saur et al., 2008). An interesting question for future research is how the maturation of fiber pathways connecting the core language network to extended networks that interact during language processing (Fedorenko et al., 2024) relates to grammar abilities in preschool children.

## 6 Conclusion

In sum, the present results highlight the role of the white matter language network for morpho-syntax acquisition during the critical preschool years between 3 and 5 years of age. The 4- to 5-year-olds, but not the 3-year-olds, showed a relationship between morpho-syntax and both dorsal pathways (to BA44 and BA6). Based on previous studies in adults, these structures may support different aspects of rule-based language processes at the word level during development, such as the application of the plural rule and mapping the plural form to motor articulation. The behavioral difference between the 3-year-olds and older children appear to be associated with developmental changes in brain structure that support grammar acquisition.

## CRediT authorship contribution statement

**Angela D. Friederici:** Writing – review & editing, Supervision, Resources, Funding acquisition, Conceptualization. **Charlotte Grosse Wiesmann:** Writing – review & editing, Supervision, Resources, Investigation, Funding acquisition, Conceptualization. **Philipp Berger:** Writing – review & editing, Supervision, Software, Methodology, Data curation. **Cheslie C. Klein:** Writing – original draft, Visualization, Validation, Methodology, Investigation, Formal analysis, Data curation, Conceptualization.

## Funding

This study was funded by the European Research Council (10.13039/100010663ERC) Starting Grant (project number REPRESENT 101117806) to CGW.

## Declaration of Competing Interest

The authors declare that they have no known competing financial interests or personal relationships that could have appeared to influence the work reported in this paper.

## Data Availability

Data will be made available on request.
