# Supplementary material for: Grammar acquisition in preschool children is related to white matter maturation of the dorsal language network
Source: Dev Cogn Neurosci. 2026 Mar 22;79:101715. doi: 10.1016/j.dcn.2026.101715 (PMC13053781; doi:10.1016/j.dcn.2026.101715)
Supplement: Supplementary file 1 — Supplementary material [file mmc1.docx]

## **Supplementary Material**

## **Grammar acquisition in preschool children is related to white matter maturation of the dorsal language network**

Cheslie C. Klein^1^*^,^*^2*^, Philipp Berger^1^*^,^*^2^, Charlotte Grosse Wiesmann^2,3⇟^ & Angela D. Friederici^1⇟^

^1^Department of Neuropsychology, Max Planck Institute for Human Cognitive and Brain Sciences, Leipzig, Germany

^2^Research Group Milestones of Early Cognitive Development, Max Planck Institute for Human Cognitive and Brain Sciences, Leipzig, Germany

^3^Cognitive Neuroscience Lab, Department of Liberal Arts and Sciences, University of Technology Nuremberg, Germany

⇟ Contributed equally

***Correspondence to:**

Cheslie C. Klein

Max Planck Institute for Human Cognitive and Brain Sciences

Department of Neuropsychology

Stephanstraße 1a, 04103 Leipzig, Germany

Email: cklein@cbs.mpg.de

**Supplementary Methods: Description of each sample of preschool aged children**

For this study, data from two samples were pooled for analyses. Sample 1 included 90 children aged 3 and 5 years (3-y.o.: N = 34, mean age = 3.63, SD = 0.24, range = 3.10 to 3.99, 12 female; 5-y.o.: N = 56, mean age = 5.48, SD = 0.47, range = 4.01 to 6.16, 27 female). Sample 2 (N = 30) included 3- and 4-year-old children (3-y.o.: N = 13, mean age = 3.26, SD = 0.18, range = 3.07 to 3.59, 8 female; 4-y.o.: N = 17, mean age = 4.32, SD = 0.18, range = 4.02 to 4.58, 8 female).

**Supplementary Figure 1: Distribution of age years per sample**


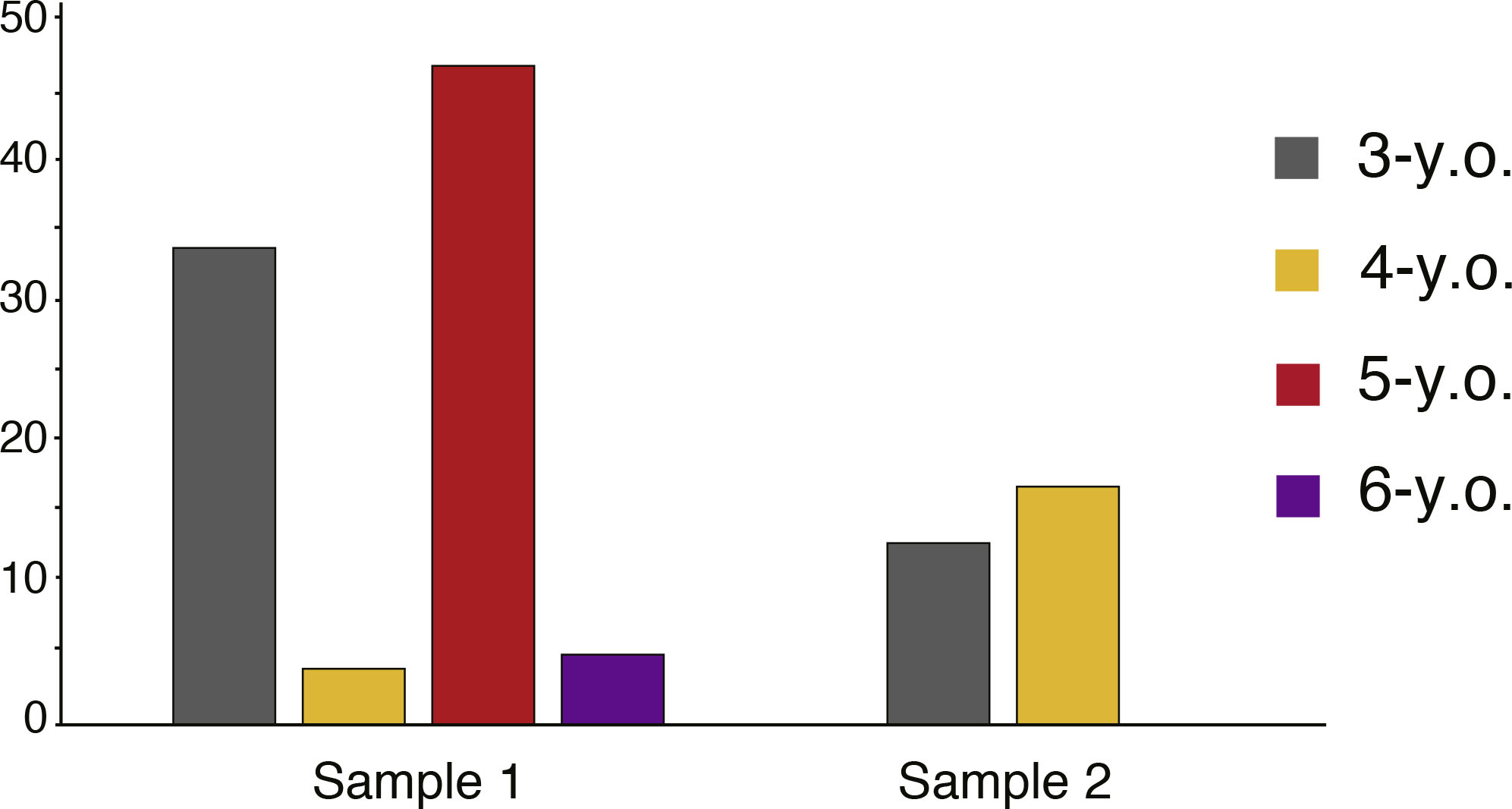


**Supplementary Table 1: Items of the morpho-syntactic word production task for each age group**

**Table 1:** Singular noun form of each item from the SETK 3-5 and its target plural form (Grimm, 2001), vowel change is indicated by capital letter.

|  | | **Singular form** | **Plural form** |
| --- | --- | --- | --- |
| (Practice item) | | Auto [engl. car] | Auto-s [engl. cars] |
| *3- to 5-y.o.: Real nouns* | | |  |
|  | Fisch [engl. fish] | | Fisch-e [engl. fish] |
|  | Schiff [engl. ship] | | Schiff-e [engl. ships] |
|  | Gabel [engl. fork] | | Gabel-n [engl. forks] |
|  | Bild [engl. picture] | | Bild-er [engl. pictures] |
|  | Stuhl [engl. chair] | | StÜhl-e [engl. chairs] |
|  | Hand [engl. hand] | | HÄnd-e [engl. hands] |
|  | Buch [engl. book] | | BÜch-er [engl. books] |
|  | Glas [engl. glass] | | GlÄs-er [engl. glasses] |
|  | Vogel [engl. bird] | | VÖgel [engl. birds] |
|  | Apfel [engl. apple] | | Äpfel [engl. apples] |
| *4- to 5-y.o.: Pseudo nouns* | | |  |
|  | Tulo | | Tulo-s |
|  | Biwo | | Biwo-s |
|  | Dolling | | Dolling-e |
|  | Ribane | | Ribane-n |
|  | Plarte | | Plarte-n |
|  | Tapsel | | Tapsel-n |
|  | Ropf | | Ropf-e/RÖpf-e |
|  | Kland | | KlÄnd-e |

**Supplementary Methods: Description of the plural noun system in German**

For plural formation, a word stem (e.g., dog) has to be combined with a plural morpheme (e.g., -s) to result in a plural word with an inherently more complex structure (e.g., dog-s) than the simple noun stem. In German, the plural of a noun can be externalized by several plural endings (i.e., –(e)n, –e, –s and –er) or no explicit marking on the noun, and additionally with a qualitative change of the stem vowel (e.g., Buch_neut_ – Büch-er [engl. book – book-s]; Werner, 1969). Thus, the German noun plural system consists of (multiple) rules depending primarily on noun phonology and gender (Köpcke, 1988). Most cases of plural nouns are covered by a few frequent rules (e.g., most female nouns take the plural marking –(e)n, as in Woche_fem_ – Woche-n [engl. week – week-s]; Köpcke, 1988). However, some irregular plural forms have to be lexicalized when no obligatory rule applies (e.g., the vowel change for masculine nouns with the plural –e, as in Ball_masc_ – Bäll-e [engl. ball – ball-s], but Tag_masc_ – Tag-e [engl. day – day-s]; Wegener, 1999). Frequent plural rules are typically acquired first during development and are also overapplied more often than less frequent rules (Kauschke, 2012).

**Supplementary Table 2: Descriptives of the morpho-syntax scores for each age group**

**Table 2:** Mean, SD, median and range of each behavioral morpho-syntax score for each age group.

|  | **Mean** | **SD** | **median** | **range** |
| --- | --- | --- | --- | --- |
| *Morpho-syntax score* | | | | |
| 3-y.o. | 0.28 | 0.89 | 0.51 | -1.76-1.16 |
| 4- to 5-y.o. | 0.16 | 0.79 | 0.12 | -1.76-1.16 |
| *Morpho-syntax score: Real nouns* | | | | |
| 3-y.o. | -0.17 | 0.88 | 0.04 | -2.02-0.78 |
| 4- to 5-y.o. | 0.44 | 0.48 | 0.59 | -2.02-0.78 |
| *Morpho-syntax score: Pseudo nouns* | | | | |
| 4- to 5-y.o. | 0.15 | 0.91 | 0.15 | -1.66-1.45 |

Note. All morpho-syntax scores were z-transformed within each age group and winsorized across age groups limiting extreme values to the 95th percentile except for the morpho-syntax score tested with real nouns which was z-transformed across both age groups. Morpho-syntax scores were z-transformed within the full behavioral sample (N = 252).

# Supplementary Figure 2: Overgeneralization errors of plural rules assigned to real and pseudo nouns in 3-, 4-, and 5-year-old children

#
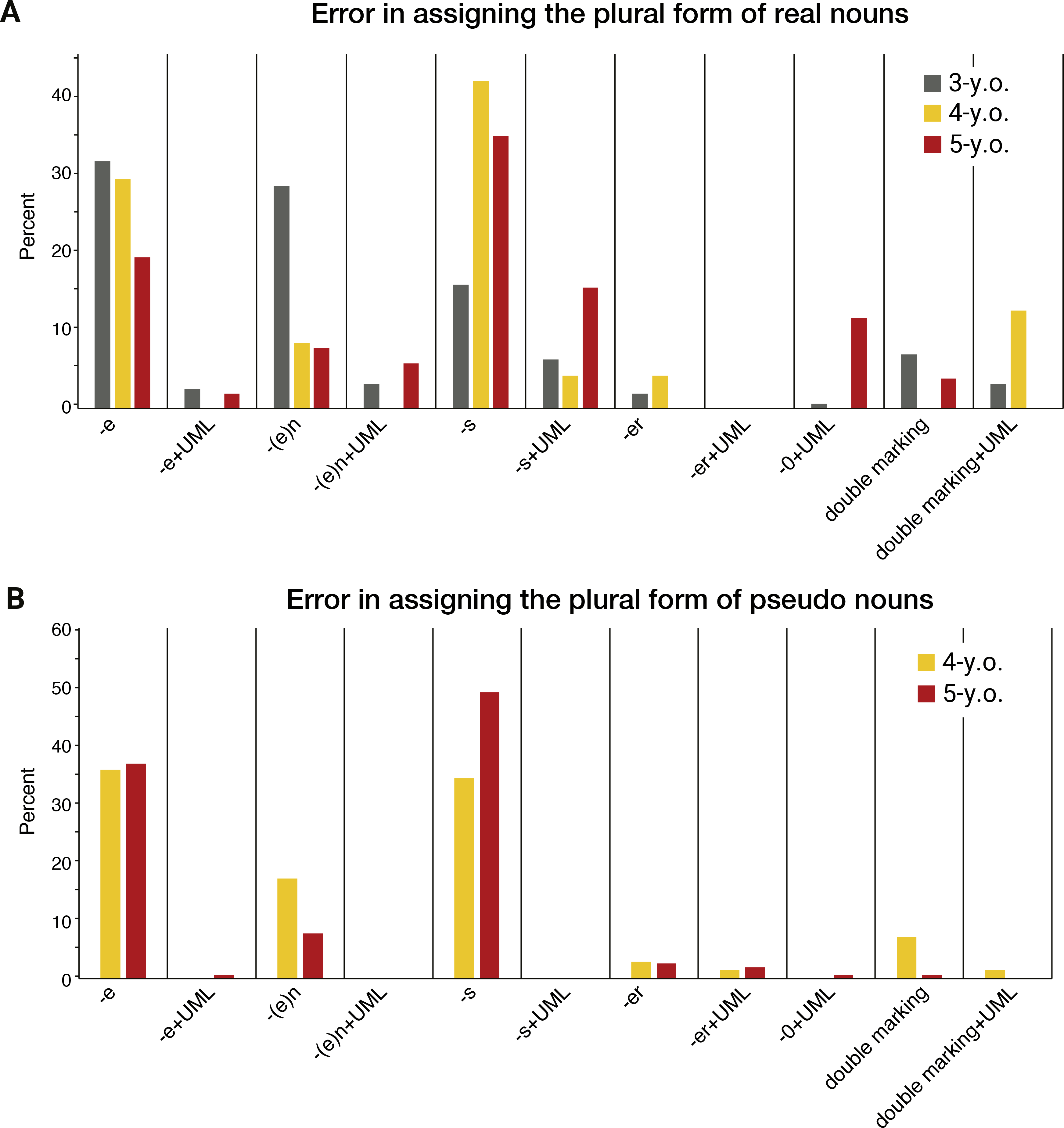


# Figure 2: Overapplication errors (in percent) of plural rules in preschool children aged 3, 4, and 5 years (N = 270). A) Percentage of errors in the assignment of plurals to real nouns (3-y.o.: gray; 4-y.o.: yellow; 5-y.o.: red). B) Percentage of errors in the assignment of plurals to pseudo nouns (4-y.o.: yellow; 5-y.o.: red).

# Supplementary Methods: Description of preregistered scores to assess children’s grammar ability on the sentence-level

We additionally preregistered two scores to assess children’s grammar ability on the sentence-level both in comprehension and production.

To receive the syntactic comprehension score, we used the subtest ‘Understanding sentences’ (orig. ‘Verstehen von Sätzen’, VS; Grimm, 2001) from the SETK 3-5 which consists of manipulation tasks. There, children are given instructions as, for example, to move objects in a certain order or to interact with another object. The items’ grammatical complexity of the presented sentence increases gradually, for instance by demanding causal relations between two actions and involving not only subject- but also object-initial sentences (VS: “Zeig mir: Der gelbe Ball, den der weiße Ball anstößt, fällt vom Tisch.” [engl. “Show me: The yellow ball bumped by the white ball falls off the table.”]; Grimm, 2001). While most items overlap in the tests for the 3- and 4- to 5-year-old children, some of the more complex constructions occur only in the older age groups as, for example, object-first subordinate clauses are not yet understood by young preschoolers (Grimm, 2001; Schipke et al., 2012). Additionally, the 3-year-olds participate in a sentence-picture-matching task in which they are asked to select one of four pictures matching the presented sentence. From this task, we then standardized the raw values within the respective age groups of the full behavioral sample (N = 252). These standardized values were then used as the syntactic comprehension score in further analyses (see Supplementary Table 3 and Figure 3).

To get an estimate for children’s syntactic production ability, we made use of the two sentence production subtests of the SETK 3-5, which were performed in the groups of 3- and 4- to 5-year-olds each. For the 3-year-old children, we used the production data from the SETK 3-5 subtest ‘Encoding semantic relations’ (orig. ‘Enkodierung semantischer Relationen’) which is a picture description task. On these pictures, people and animals perform an action with spatial relation to an object eliciting the use of prepositional phrases with varying degrees of difficulty depending on the required preposition (Grimm, 1975). For the 4- to 5-year-olds, we selected the subtest ‘Sentence memory’ (orig. ‘Satzgedächtnis’, SG), in which children are asked to reproduce sentences consisting of six to ten words with correct morpho-syntactic inflection and either plausible (SG: “Lena lacht, nachdem sie gekitzelt wurde.” [engl. “Lena laughs after being tickled.”]; Grimm, 2001) or implausible meaning (SG: “Ein frecher Fußball, der den alten Kasper heiratet, ist müde.” [engl. “A cheeky soccer ball marrying the old Punch is tired.”]; Grimm, 2001). The length of the sentences is determined, so that they cannot be solely retrieved from the child’s working memory but require reconstruction of the sentence structure using the child’s grammatical knowledge (Grimm, 2001). This effect is further enhanced by sentences with implausible meaning, as children cannot rely on their real-world knowledge (Grimm, 2001). To receive the syntactic production score, we recoded the production data to estimate the longest syntactically correct fragment in words per items as described in Klein et al. (2023). Then, we standardized these values within the age groups to account for differences in the respective tasks for 3- and 4- to 5-year-old children which were used in further analyses (see Supplementary Table 3 and Figure 3).

# Supplementary Methods: Description of preregistered score to assess children’s general language ability

To investigate the relation between children’s general language ability and brain structure, we preregistered an aggregated score from the SETK 3-5 as described in Klein et al. (2023). For this, we standardized the raw values of each subtest within the age groups of each sample to account for differences in the item structure of the test version for 3- versus 4- to 5-year-olds. Then, we combined these z-scores to the respective scale for language comprehension, production or memory as defined by the SETK 3-5 (Grimm, 2001) and summed these scale values to the general language score (see Supplementary Table 3 and Figure 3).

**Supplementary Table 3: Descriptives of the additional language scores for each age group**

**Table 3:** Mean, SD, median and range of each additional language score for each age group.

|  | **Mean** | **SD** | **median** | **range** |
| --- | --- | --- | --- | --- |
| *Syntactic comprehension score* | | | | |
| 3-y.o. | 0.38 | 0.86 | 0.51 | -1.24-1.56 |
| 4- to 5-y.o. | 0.25 | 0.75 | 0.10 | -1.24-1.56 |
| *Syntactic production score* | | | | |
| 3-y.o. | 0.17 | 0.76 | 0.26 | -1.05-1.15 |
| 4- to 5-y.o. | 0.36 | 0.59 | 0.52 | -1.05-1.15 |
| *General language score* | | | | |
| 3-y.o. | 0.44 | 0.91 | 0.65 | -1.25-1.52 |
| 4- to 5-y.o. | 0.31 | 0.73 | 0.41 | -1.25-1.52 |

Note. All language scores were z-transformed within each age group of the full behavioral sample (N = 252) and winsorized across age groups limiting extreme values to the 95th percentile.

# Supplementary Figure 3: Distribution of the additional language scores per age group

#
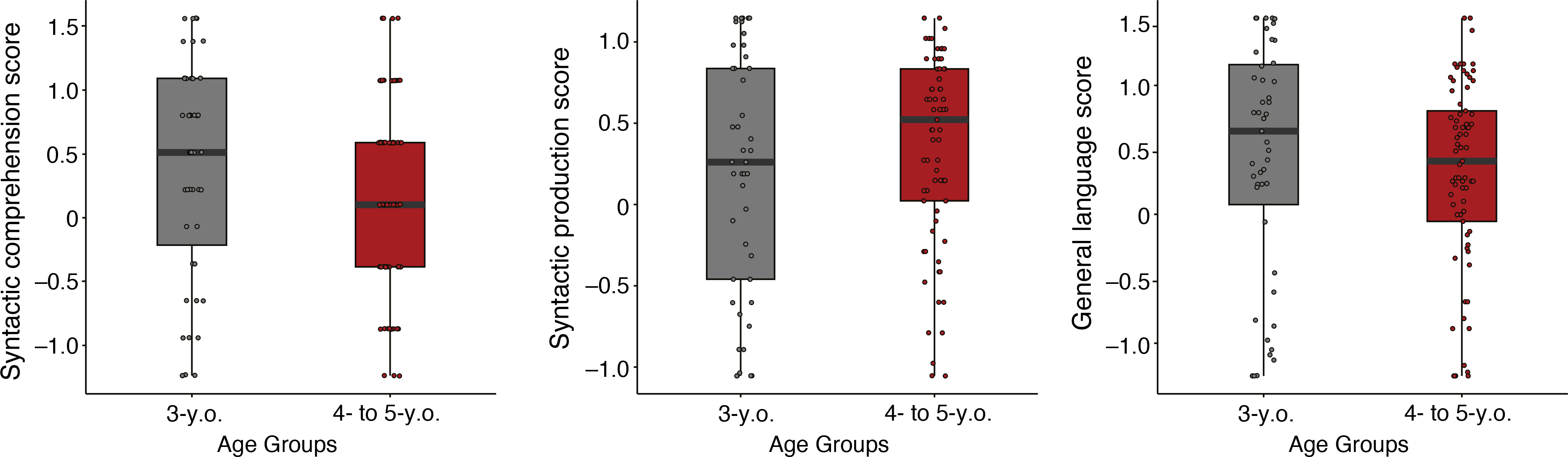


**Figure 3:** Distribution of z-transformed additional language scores (3-y.o.: grey; 4- to 5-y.o.: red). All language scores were standardized within each age group and winsorized across age groups limiting extreme values to the 95th percentile.

**Supplementary Table 4: Summary of the parameters for MRI acquisition in both samples**

**Table 4:** Scanning parameters used for acquisition of diffusion- and T1-weighted MRI data separated by samples, values for each parameter of the 3- & 5-year-old, and 3- & 4-year-old children.

| **Scanning parameter** | **Sample 1: 3- & 5-y.o.** | **Sample 2: 3- & 4-y.o.** | |
| --- | --- | --- | --- |
| **Head coil** | 12-channel | 32-channel | |
| *Diffusion-weighted MRI* | | |  |
| **sequence** | optimized monopolar  Stejskal-Tanner EPI | multiplexed EPI | |
| **TR** | 8,000 ms | 4,000 ms | |
| **TE** | 83.0 ms | 75.4 ms | |
| **b-value** | 1,000 s/mm^2^ | 1,000 s/mm^2^ | |
| **directions** | 60 | 60 | |
| **voxel size** | 1.86×1.86×1.9 mm | 1.9 mm isotropic | |
| *T1-weighted MRI* | | |  |
| **sequence** | MP2RAGE | MP2RAGE | |
| **TR** | 5,000 ms | 5,000 ms | |
| **TE** | 2.82 ms | 3.24 ms | |
| **TI_1_/TI_2_** | 700 ms/2,500 ms | 700 ms/2,500 ms | |
| **α_1_/α_2_** | 4°/5° | 4°/5° | |
| **voxel size** | 1.3 mm isotropic | 1.2×1.0×1.0 mm | |

# Supplementary Figure 4: Distribution of absolute head motion during scanning per age group

#
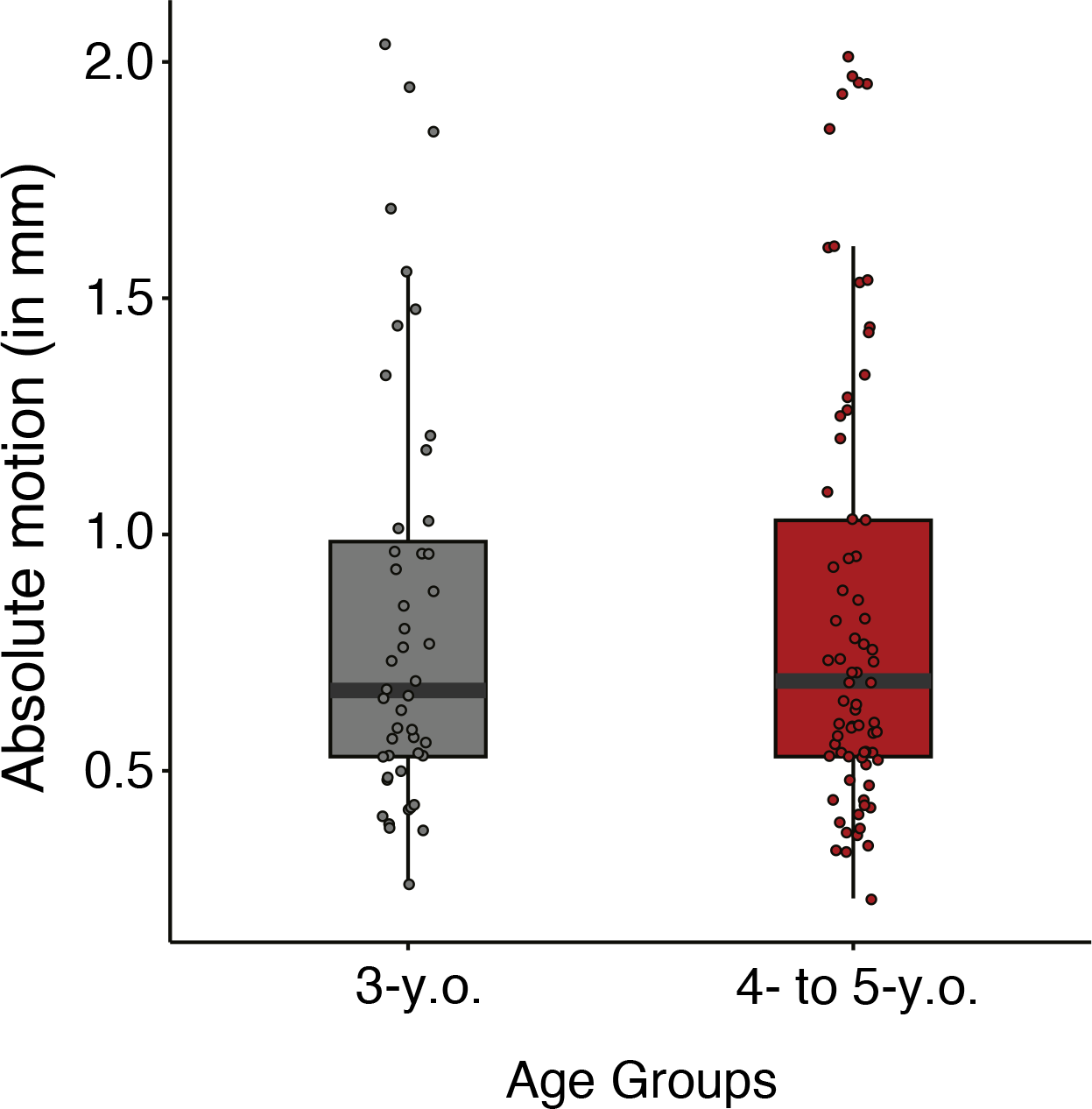


**Figure 4:** Distribution of absolute head motion in the scanner (3-y.o.: grey; 4- to 5-y.o.: red).

# Supplementary Figure 5: Masks used for segmentation to disentangle the two dorsal fiber pathways

**Figure 5:** A) Masks used for segmentation of the dorsal fiber pathway targeting BA44 to include streamlines (green) based on prior anatomical assumptions (Frey et al., 2008; Glasser & Rilling, 2008). B) Masks used for segmentation of the dorsal fiber pathway targeting BA6 to include (green) and exclude (red) streamlines. Probability map of the arcuate fascicle (heat map) as provided by pyAFQ (Kruper et al., 2021) was used for refinement of both fiber pathways.

**Supplementary Table 5: List of ROIs used for fiber pathway segmentation**

**Table 5:** Start, end, and waypoint ROIs, as well as exclusion ROIs, for each fiber pathway, whether fibers were allowed to cross the midline, and the probability map used to refine each pathway.

|  | **Dorsal pathway to BA44** | **Dorsal pathway**  **to BA6** | **IFOF** | **Corticospinal tract** |
| --- | --- | --- | --- | --- |
| *Start ROI* | Pars opercularis  (label index 11)* | Precentral gyrus  (label index 1)* | default for IFOF | default for CST |
| *End ROI* | default for AF | default for AF | default for IFOF | default for CST |
| *Waypoint ROIs* | default for AF  Pars opercularis  (label index 5)† | default for AF  Precentral gyrus  (label index 6)† | default for IFOF | default for CST |
| *Exclusion ROIs* | -  (default for AF) | Pars opercularis  (label index 5)†  default end ROI for CST | default for IFOF | -  (default for CST) |
| *Allowed to cross midline* | no | no | no | no |
| *Probability map* | default for AF | default for AF | default for IFOF | default for CST |

Note. We relied on the default ROIs provided by pyAFQ (Kruper et al., 2021) for the segmentation of the IFOF and corticospinal tract (CST). To determine the termination points of the dorsal pathways in BA44 and BA6, we modified the default pyAFQ pipeline for the arcuate fascicle (AF). *Labels were obtained from the AAL atlas provided by pyAFQ (Rokem, 2021). †Labels were used from the Harvard-Oxford atlas (Makris et al., 2006).

# Supplementary Figure 6: Examples for inclusion and exclusion criteria of the dorsal fiber pathways after manual quality check of segmentation results

**Figure 6:** Inclusion criteria for segmentation of the two dorsal fiber pathways as the frontal part of both pathways terminate in distinct regions of BA44 and BA6, and two examples of excluded fiber pathways as streamlines were spurious or followed an anatomically implausible path, or frontal endpoints of pathways did not lead to distinct termination in the respective target regions of BA44 and BA6. Modified from Eichner, C., Berger, P., Klein, C. C., & Friederici, A. D. (2024). Lateralization of dorsal fiber tract targeting Broca’s area concurs with language skills during development. Progress in Neurobiology, 102602, https://doi.org/10.1016/j.pneurobio.2024.102602.

**Supplementary Table 6: Overview of the relation between morpho-syntax scores and fiber pathways in the left hemisphere for the pooled sample**

**Table 6:** Significant relations between morpho-syntax scores and fiber pathways in the pooled sample, by measure and age group.

| **Pooled sample** | **Fiber pathway** | **Measure** | **Cluster size** |
| --- | --- | --- | --- |
| *Morpho-syntax score* | | |  |
| 3-y.o. + 4- to 5-y.o. | n.s. | - | - |
| 3-y.o. × 4- to 5-y.o. | Dorsal pathway to BA6  Ventral pathway | RD  FA, Streamline count | 17  11, - |
| 3-y.o. | n.s. | - | - |
| 4- to 5-y.o. | Dorsal pathway to BA6  Ventral pathway | RD  Streamline count | 17  - |
| *Morpho-syntax score: Real nouns* | | |  |
| 3-y.o. + 4- to 5-y.o. | n.s. | - | - |
| 3-y.o. × 4- to 5-y.o. | Dorsal pathway to BA44  Dorsal pathway to BA6 | RD  MD, RD | 16  26, 18 |
| 3-y.o. | n.s. | - | - |
| 4- to 5-y.o. | Dorsal pathway to BA44  Dorsal pathway to BA6 | MD, RD  MD, RD | 31, 18  31, 18 |
| *Morpho-syntax score: Pseudo nouns* | | |  |
| 4- to 5-y.o. | Ventral pathway | Streamline count | - |

# Supplementary Results: Preregistered analyses on the relation between preschoolers’ streamline count and their morpho-syntactic abilities

# Beside the node-based analyses, we further preregistered total streamline count per tract as a measure for maturation. In all models testing for a main effect, we performed one-sided tests as we expected streamline count to increase with maturation and higher cognitive function (Lebel & Deoni, 2018). Since streamline count constitutes one value per fiber pathway, the alpha-level was set at *p* = 0.05.

# For the morpho-syntax score, we found an age interaction with streamline count in the ventral pathway (β = 407.1, SE = 158.3, F^2^ = 0.06, *t*(108) = 2.572, *p* = 0.012). When testing each age group separately, we further observed an effect in the 4- to 5-year-old children (β = 258.7, SE = 111.3, F^2^ = 0.08, *t*(63) = 4.494, *p* = 0.012), but not the 3-year-olds. In the exploratory analyses, we found that morpho-syntax scores tested only with pseudo nouns correlated with streamline count in the ventral tract in the 4- to 5-year-olds (β = 190.2, SE = 99.8, F^2^ = 0.05, *t*(62) = 1.905, *p* = 0.031).

**Supplementary Table 7: Overview of the relation between morpho-syntax scores and fiber pathways in the left hemisphere for Sample 1**

**Table 7:** Significant relations between morpho-syntax scores and fiber pathways for Sample 1, by measure and age group.

| **Sample 1** | **Fiber pathway** | **Measure** | **Cluster size** |
| --- | --- | --- | --- |
| *Morpho-syntax score* | | |  |
| 3-y.o. + 5-y.o. | n.s. | - | - |
| 3-y.o. × 5-y.o. | Dorsal pathway to BA6  Ventral pathway | RD  FA, RD, Streamline count | 15  14, 12, - |
| 3-y.o. | n.s. | - | - |
| 5-y.o. | Dorsal pathway to BA6  Ventral pathway | FA, RD  FA, Streamline count | 14, 16  17, - |
| *Morpho-syntax score: Real nouns* | | |  |
| 3-y.o. + 5-y.o. | n.s. | - | - |
| 3-y.o. × 5-y.o. | Dorsal pathway to BA44  Dorsal pathway to BA6  Ventral pathway | FA, RD  MD, RD  FA, Streamline count | 9, 17  20, 18  13, - |
| 3-y.o. | n.s. | - | - |
| 5-y.o. | Dorsal pathway to BA44  Dorsal pathway to BA6 | RD  RD | 20  15 |
| *Morpho-syntax score: Pseudo nouns* | | |  |
| 5-y.o. | n.s. | - | - |

**Supplementary Table 8: Overview of the relation between morpho-syntax scores and fiber pathways in the left hemisphere for Sample 2**

**Table 8:** Significant relations between morpho-syntax scores and fiber pathways for Sample 2, by measure and age group.

| **Sample 2** | **Fiber pathway** | **Measure** | **Cluster size** |
| --- | --- | --- | --- |
| *Morpho-syntax score* | | |  |
| 3-y.o. + 4-y.o. | n.s. | - | - |
| 3-y.o. × 4-y.o. | Dorsal pathway to BA44  Dorsal pathway to BA6 | MD, RD  MD | 20, 20  28 |
| 3-y.o. | n.s. | - | - |
| 4-y.o. | Dorsal pathway to BA44  Dorsal pathway to BA6 | MD, RD  MD | 27, 11  20 |
| *Morpho-syntax score: Real nouns* | | |  |
| 3-y.o. + 4-y.o. | n.s. | - | - |
| 3-y.o. × 4-y.o. | Dorsal pathway to BA6 | FA, MD, RD | 11, 34, 14 |
| 3-y.o. | n.s. | - | - |
| 4-y.o. | Dorsal pathway to BA44 | MD | 17 |
| *Morpho-syntax score: Pseudo nouns* | | |  |
| 4-y.o. | Dorsal pathway to BA44 | RD | 12 |

# Supplementary Results: Analyses on the relation between preschoolers’ grammar ability on the word-level and language pathways within each sample

In the main manuscript, we present the analyses of a pooled data set from two samples. Sample 1 included children aged 3 and 5 years, and Sample 2 included children aged 3 and 4 years (see *Supplementary Methods* and *Supplementary Figure 1* for details on the age groups). Additionally, we conducted separate analyses within each sample.

In both samples, younger children at the age of 3 years differed from older children aged 4 and 5 years. In Sample 1, there was no significant main effect of children’s morpho-syntax scores on their brain maturational measures in the reconstructed pathways, but a significant interaction with age group. This age interaction was found in the dorsal pathway to BA6 (anterior part: RD, node range = 17-31, cluster size = 15, cluster threshold = 14, β = -0.03, SE = 0.01, F^2^ = 0.12, df *=* 78, *p* < 0.017 FWE-corrected) and in the ventral pathway (streamline count, β = 413.4, SE = 181.5, F^2^ = 0.06, *t*(80) = 2.278, *p* = 0.025; central part: FA, node range = 39-52, cluster size = 14, cluster threshold = 10, β = 0.032, SE = 0.007, F^2^ = 0.24, df = 80, *p* < 0.017 FWE-corrected; RD, node range = 40-51, cluster size = 12, cluster threshold = 12, β = -0.031, SE = 0.010, F^2^ = 0.13, df = 80, *p* < 0.017 FWE-corrected), but not the dorsal pathway to BA44. No main effect or interaction with age was found in the corticospinal tract serving as control tract. To follow-up on these interactions, we analyzed the two age groups, i.e. the 3- and 5-year-olds, separately. This revealed that the 5-year-old children showed a significant relation between their morpho-syntax scores and brain maturational measures in the dorsal pathway to BA6 (anterior part: FA, node range = 21-34, cluster size = 14, cluster threshold = 11, β = 0.023, SE = 0.008, F^2^ = 0.13, df = 47, *p* < 0.017 FWE-corrected; RD, node range = 18-33, cluster size = 16, cluster threshold = 14, β = -0.020, SE = 0.007, F^2^ = 0.12, df = 47, *p* < 0.017 FWE-corrected) and the ventral pathway (streamline count, β = 322.0, SE = 136.3, F^2^ = 0.12, *t*(48) = 2.363, *p* = 0.011; central part: FA, node range = 42-58, cluster size = 17, cluster threshold = 11, β = 0.021, SE = 0.065, F^2^ = 0.19, df = 48, *p* < 0.017 FWE-corrected). No effect was found in the dorsal pathway to BA44 or the control tract in the 5-year-olds. The 3-year-olds did not show any significant relation in either of the three language-related fiber pathways or the control tract.

In Sample 2, again no main effect of children’s morpho-syntax scores on their language-related pathways was found. Further, we also found an interaction with age group in the dorsal pathway to BA6, but in a different spatial location than in Sample 1 (part terminating in the temporal lobe: MD, node range = 73-100, cluster size = 28, cluster threshold = 20, β = -0.035, SE = 0.009, F^2^ = 0.68, df = 22, *p* < 0.017 FWE-corrected). Additionally, we found an age interaction in the dorsal pathway to BA44 (posterior part: MD, node range = 71-90, cluster size = 20, cluster threshold = 18, β = -0.033, SE = 0.010, F^2^ = 0.55, df = 22, *p* < 0.017 FWE-corrected; anterior part: RD, node range =18-37, cluster size = 20, cluster threshold = 12, β = -0.046, SE = 0.011, F^2^ = 0.81, df = 22, *p* < 0.017 FWE-corrected). No interaction with age was found in the ventral pathway, nor the control tract. Follow-up analyses separated by age group revealed the 4-year-old children, but not the 3-year-olds, showed a significant relation in the dorsal pathway to BA6 (anterior part: MD, node range = 10-29, cluster size = 20, cluster threshold = 19, β = -0.022, SE = 0.006, F^2^ = 1.28, df = 11, *p* < 0.017 FWE-corrected) and in the dorsal pathway to BA44 (anterior part: MD, node range = 7-33, cluster size = 27, cluster threshold = 16, β = -0.017, SE = 0.004, F^2^ = 0.98, df = 11, *p* < 0.017 FWE-corrected; RD, node range = 27-37, cluster size = 11, cluster threshold = 11, β = -0.023, SE = 0.007, F^2^ = 0.69, df = 11, *p* < 0.017 FWE-corrected). In line with the absence of an interaction, no significant effect was found in the ventral pathway or the corticospinal tract in either of the two age groups.

For children’s real noun morpho-syntax scores, as before, no significant main effect but significant interactions with age group were found in both samples. In Sample 1, the interaction was found in the dorsal pathway to BA6 (anterior part: MD, node range = 7-26, cluster size = 20, cluster threshold = 20, β = -0.026, SE = 0.007, F^2^ = 0.16, df = 77, *p* < 0.017 FWE-corrected; RD, node range =16-33, cluster size = 18, cluster threshold = 13, β = -0.041, SE = 0.011, df = 77, *p* < 0.017 FWE-corrected), in the dorsal pathway to BA44 (anterior to central part: FA, node range = 29-37, cluster size = 9, cluster threshold = 9, β = 0.033, SE = 0.010, F^2^ = 0.13, df = 75, *p* < 0.017 FWE-corrected; RD, node range =28-44, cluster size = 17, cluster threshold = 12, β = -0.041, SE = 0.012, F^2^ = 0.16, df = 75, *p* < 0.017 FWE-corrected), and the ventral pathway (streamline count, β = 453.4, SE = 213.0, F^2^ = 0.06, *t*(79) = 2.128, *p* = 0.036; central part: FA, node range = 40-52, cluster size = 13, cluster threshold = 10, β = 0.034, SE = 0.009, F^2^ = 0.18, df = 79, *p* < 0.017 FWE-corrected). As before, follow-up analyses revealed that the 5-year-olds, but not the 3-year-olds, showed an effect in the dorsal pathway to BA6 (anterior part: RD, node range = 18-32, cluster size = 15, cluster threshold = 15, β = -0.031, SE = 0.009, F^2^ = 0.19, df = 47, *p* < 0.017 FWE-corrected) and additionally in the dorsal pathway to BA44 (anterior to central part: RD, node range = 25-44, cluster size = 20, cluster threshold = 13, β = -0.028, SE = 0.009, F^2^ = 0.19, df = 45, *p* < 0.017 FWE-corrected). No effect was found for the ventral pathway in either of the two age groups.

In Sample 2, the interaction was also located in the dorsal pathway to BA6 (anterior to central part: FA, node range = 35-45, cluster size = 11, cluster threshold = 11, β = 0.073, SE = 0.018, F^2^ = 0.74, df = 22, *p* < 0.017 FWE-corrected; MD, node range = 11-44, cluster size = 34, cluster threshold = 18, β = -0.047, SE = 0.014, F^2^ = 0.52, df = 22, *p* < 0.017 FWE-corrected; RD, node range =32-45, cluster size = 14, cluster threshold = 12, β = -0.074, SE = 0.016, F^2^ = 0.94, df *=* 22, *p* < 0.017 FWE-corrected). No significant interaction was found in the dorsal pathway to BA44 or the ventral pathway in this sample. Following-up on this interaction, 4-year-old children did not show a significant effect in the dorsal pathway to BA6, but a significant relation in the dorsal pathway to BA44 (anterior part: MD, node range = 27-43, cluster size = 17, cluster threshold = 17, β = -0.026, SE = 0.008, F^2^ = 0.96, df = 11, *p* < 0.017 FWE-corrected). No effect was found for the ventral pathway. No significant effect for the real noun morpho-syntax scores was found in the 3-year-olds in either sample. Moreover, no main effect or interaction was found for the control tract.

Further, we exploratorily tested for a relation between children’s pseudo noun morpho-syntax scores and brain structure within each sample. There, we found a significant relation with RD in the dorsal pathway to BA44 in the 4-year-olds in Sample 2 (anterior part: node range = 26-37, cluster size = 12, cluster threshold = 11, β = -0.027, SE = 0.006, F^2^ = 1.63, df = 11, *p* < 0.017 FWE-corrected). No effect was found in the dorsal pathway to BA6, the ventral pathway or the control tract. In Sample 1, the 5-year-old children showed no effect of their pseudo noun morpho-syntax scores in any language-related fiber pathway or the control tract.

# Supplementary Results: Analyses on the relation between the additional language scores and language pathways

# We aimed to examine the relation between preschoolers’ grammar ability on the sentence-level and the maturation of language fiber pathways. For this, we preregistered two scores from a sentence comprehension and production task, and correlated them with children’s brain structural measures of the dorsal pathway to BA44, the dorsal pathway to BA6, the ventral pathway, and the corticospinal tract as a control. We controlled for sex, non-verbal IQ, handedness, eTIV, and additionally for family history of dyslexia and sample. Multiple comparison correction was applied at *p* = 0.05 with a further Bonferroni-correction of N = 3 microstructural measures.

# When testing for a relation between children’s syntactic comprehension and production scores and brain structure, we found no main effect or interaction in any language fiber pathway nor the control tract. Further, no significant effect in any language fiber pathway or the control tract was found for either age group.

In addition to our investigation on preschooler’s grammar ability, we further assessed the relation between their general language ability and the maturation of language fiber pathways. In a preregistered procedure, we related children’s overall performance in the general language test to brain structural measures in the three language pathways and the control tract. We found a significant main effect, including both age groups, in the control tract (FA, node range = 22-38, cluster size = 17, cluster threshold = 14, β = 0.014, SE = 0.004, F^2^ = 0.13, df = 110, *p* < 0.017 FWE-corrected), but not in the language pathways. We found no significant interaction with age group and no significant effect in any age group.

When exploratorily testing for a relation between children’s pseudo noun morpho-syntax scores and brain structure, we found no significant relation with the microstructural brain measures in any language-related fiber pathway or the control tract.

# Supplementary Results: Exploratory analyses on preschooler’s morpho-syntactic ability and language pathways in the right hemisphere

In addition to our main analyses, we examined the relationship between children’s morpho-syntax scores and brain measures of the three homologues language pathways (i.e., the dorsal pathway to BA44 and BA6, and IFOF) and control tract (i.e., the corticospinal tract) in the right hemisphere. This was done to test whether preschoolers exhibit hemisphere-specific differences in the association between brain structures and morpho-syntactic ability, given the left-lateralization of language in the adult brain (Toga & Thompson, 2003).

Consistent with the findings in the left hemisphere, no significant main effect was observed between children’s morpho-syntax scores and brain measures in the right fiber pathways. Instead, there was a significant interaction with age group in the right dorsal pathway to BA6 (anterior to central part: MD, node range = 23-50, cluster size = 28, cluster threshold = 20, β = -0.022, SE = 0.006, F^2^ = 0.12, df = 96, *p* < 0.017 FWE-corrected; RD, node range = 29-50, cluster size = 22, cluster threshold = 13, β = -0.021, SE = 0.007, F^2^ = 0.10, df = 96, *p* < 0.017 FWE-corrected), the right dorsal pathway to BA44 (central part: MD, node range = 43-60, cluster size = 18, cluster threshold = 16, β = -0.018, SE = 0.006, F^2^ = 0.10, df = 103, *p* < 0.017 FWE-corrected), and the right ventral pathway (streamline count, β = 500.7, SE = 141.5, F^2^ = 0.11, *t*(109) = 3.538, *p* < 0.001; posterior to central part: FA, node range = 30-43, cluster size = 14, cluster threshold = 11, β = 0.027, SE = 0.007, F^2^ = 0.15, df = 109, *p* < 0.017 FWE-corrected; anterior part: MD, node range = 78-93, cluster size = 16, cluster threshold = 16, β = -0.019, SE = 0.007, F^2^ = 0.07, df = 109, *p* < 0.017 FWE-corrected; central part: RD, node range = 31-57, cluster size = 27, cluster threshold = 13, β = -0.025, SE = 0.008, F^2^ = 0.09, df = 109, *p* < 0.017 FWE-corrected). This is similar to the results in the left hemisphere, but notably most effects were found in the central to posterior parts of the right-hemispheric language pathways. Analyses by age group revealed that the 4- to 5-year-old children showed a significant relation between morpho-syntax scores and the right ventral pathway (streamline count, β = 262.9, SE = 112.8, F^2^ = 0.11, *t*(64) = 2.330, *p* = 0.011; anterior to central part: RD, node range = 66-85, cluster size = 20, cluster threshold = 14, β = -0.017, SE = 0.005, F^2^ = 0.27, df = 64, *p* < 0.017 FWE-corrected), but not with the dorsal pathway to BA6 or BA44. 3-year-olds did not show a significant effect in any of the right language pathways, paralleling the findings in the left hemisphere. No significant main effect or interaction was found for the control tract (i.e., the right corticospinal tract).

**Supplementary Results: Additional analyses controlling for absolute head motion**

To investigate if differences in motion was driving the observed differences between the 3-year-old children and the two older age groups in each sample, we conducted additional analyses testing for an interaction but additionally controlling for children’s absolute head motion in the scanner. These analyses were performed on the mean MD or RD of the respective significant cluster and revealed that the observed interactions with age group were not driven by motion in either sample. The age interaction remained significant for the morpho-syntax scores tested with real nouns in the dorsal pathway to BA6 (MD: β = -0.025, SE = 0.008, *t*(106) = -3.281, *p* = 0.001; RD: β = -0.036, SE = 0.010, *t*(106) = -3.474, *p* < 0.001) and the dorsal pathway to BA44 (RD: β = -0.036, SE = 0.012, *t*(104) = -3.218, *p* = 0.002) when additionally controlling for motion.

**Supplementary Results: Sensitivity analysis to estimate the required effect size given the sample size of the 3-year-olds**

We observed consistent differences between the 3-year-old children and the older age group, with main effects found only in the 4- to 5-year-olds. However, the sample size of the 3-year-olds was smaller compared to the older age group (47 vs. 73 children). To investigate whether this difference in sample size substantially influenced our results, we performed a post-hoc sensitivity analysis using G*Power (version 3.1).

Based on the sample size of the 3-year-olds (N = 47) and a desired power of 80%, the estimated required effect size for the linear regression model was F^2^ = 0.18. Notably, in the older preschoolers, we observed similar to larger effect sizes for the relation between their morpho-syntax scores tested with real nouns and brain microstructural measures: F^2^ = 0.18 for MD and F^2^ = 0.19 for RD in the dorsal tract to BA44, and F^2^ = 0.21 for MD and F^2^ = 0.22 for RD in the dorsal tract to BA6.

# Supplementary References

Frey, S., Campbell, J. S. W., Pike, G. B., & Petrides, M. (2008). Dissociating the Human Language Pathways with High Angular Resolution Diffusion Fiber Tractography. *Journal of Neuroscience*, *28*(45), 11435–11444. https://doi.org/10.1523/JNEUROSCI.2388-08.2008

Glasser, M. F., & Rilling, J. K. (2008). DTI Tractography of the Human Brain’s Language Pathways. *Cerebral Cortex*, *18*(11), 2471–2482. https://doi.org/10.1093/cercor/bhn011

Grimm, H. (1975). On the Child’s Acquisition of Semantic Structure Underlying the Wordfield of Prepositions. *Language and Speech*, *18*(2), 97–119. https://doi.org/10.1177/002383097501800201

Grimm, H. (2001). *Sprachentwicklungstest für drei- bis fünfjährige Kinder: SETK 3–5. Diagnose von Sprachverarbeitungsfähigkeiten und auditiven Gedächtnisleistungen*. Hogrefe: Verlag für Psychologie.

Kauschke, C. (2012). *Kindlicher spracherwerb im deutschen: Verläufe, forschungsmethoden, erklärungsansätze*. De Gruyter.

Klein, C. C., Berger, P., Goucha, T., Friederici, A. D., & Grosse Wiesmann, C. (2023). Children’s syntax is supported by the maturation of BA44 at 4 years, but of the posterior STS at 3 years of age. *Cerebral Cortex*, *33*(9), 5426–5435. https://doi.org/10.1093/cercor/bhac430

Köpcke, K.-M. (1988). Schemas in German plural formation. *Lingua*, *74*(4), 303–335. https://doi.org/10.1016/0024-3841(88)90064-2

Kruper, J., Yeatman, J. D., Richie-Halford, A., Bloom, D., Grotheer, M., Caffarra, S., Kiar, G., Karipidis, I. I., Roy, E., Chandio, B. Q., Garyfallidis, E., & Rokem, A. (2021). Evaluating the Reliability of Human Brain White Matter Tractometry. *Aperture Neuro*, *2021*(1), 25. https://doi.org/10.52294/e6198273-b8e3-4b63-babb-6e6b0da10669

Lebel, C., & Deoni, S. (2018). The development of brain white matter microstructure. *NeuroImage*, *182*, 207–218. https://doi.org/10.1016/j.neuroimage.2017.12.097

Makris, N., Goldstein, J. M., Kennedy, D., Hodge, S. M., Caviness, V. S., Faraone, S. V., Tsuang, M. T., & Seidman, L. J. (2006). Decreased volume of left and total anterior insular lobule in schizophrenia. *Schizophrenia Research*, *83*(2–3), 155–171. https://doi.org/10.1016/j.schres.2005.11.020

Rokem, A. (2021). *AAL atlas for Automated Fiber Quantification* (p. 237315 Bytes) [Dataset]. figshare. https://doi.org/10.6084/M9.FIGSHARE.14787504.V1

Schipke, C. S., Knoll, L. J., Friederici, A. D., & Oberecker, R. (2012). Preschool children’s interpretation of object-initial sentences: Neural correlates of their behavioral performance: Children’s interpretation of object-initial sentences. *Developmental Science*, *15*(6), 762–774. https://doi.org/10.1111/j.1467-7687.2012.01167.x

Toga, A. W., & Thompson, P. M. (2003). Mapping brain asymmetry. *Nature Reviews Neuroscience*, *4*(1), 37–48. https://doi.org/10.1038/nrn1009

Wegener, H. (1999). Die Pluralbildung im Deutschen—Ein Versuch im Rahmen der Optimalitätstheorie. *Linguistik Online*, *4*(3). https://doi.org/10.13092/lo.4.1032

Werner, O. (1969). Das deutsche Pluralsystem (Strukturelle Diachronie). In *Sprache, Gegenwart und Geschichte: Probleme der Synchronie und Diachronie: Jahrbuch 1968* (pp. 92–128).
